# Bidirectional f-Divergence-Based Deep Generative Method for Imputing Missing Values in Time-Series Data

**DOI:** 10.3390/stats8010007

**Published:** 2025-01-14

**Authors:** Wen-Shan Liu, Tong Si, Aldas Kriauciunas, Marcus Snell, Haijun Gong

**Affiliations:** 1 Department of Health and Clinical Outcomes Research, Saint Louis University, St. Louis, MO 63103, USA; 2 Department of Mathematics and Computer Science, Culver-Stockton College, Canton, MO 63435, USA; 3 Department of Mathematics and Statistics, Saint Louis University, St. Louis, MO 63103, USA

**Keywords:** missing value imputation, time series, generative adversarial network, f-divergence, bidirectional gated recurrent unit

## Abstract

Imputing missing values in high-dimensional time-series data remains a significant challenge in statistics and machine learning. Although various methods have been proposed in recent years, many struggle with limitations and reduced accuracy, particularly when the missing rate is high. In this work, we present a novel f-divergence-based bidirectional generative adversarial imputation network, tf-BiGAIN, designed to address these challenges in time-series data imputation. Unlike traditional imputation methods, tf-BiGAIN employs a generative model to synthesize missing values without relying on distributional assumptions. The imputation process is achieved by training two neural networks, implemented using bidirectional modified gated recurrent units, with f-divergence serving as the objective function to guide optimization. Compared to existing deep learning-based methods, tf-BiGAIN introduces two key innovations. First, the use of f-divergence provides a flexible and adaptable framework for optimizing the model across diverse imputation tasks, enhancing its versatility. Second, the use of bidirectional gated recurrent units allows the model to leverage both forward and backward temporal information. This bidirectional approach enables the model to effectively capture dependencies from both past and future observations, enhancing its imputation accuracy and robustness. We applied tf-BiGAIN to analyze two real-world time-series datasets, demonstrating its superior performance in imputing missing values and outperforming existing methods in terms of accuracy and robustness.

## 1. Introduction

Time-series data are widely collected across various fields, including climate and weather prediction [1,2], economic and financial forecasting [3,4], medicine and healthcare or clinical analytics [5,6], traffic flow monitoring [7,8], the analysis of microarray or single-cell transcriptomics data [9–11], and the internet of things [12]. Unlike static datasets, time-series data encapsulate temporal dependencies, making them indispensable for dynamic predictive modeling and analysis, for example, genetic network reconstruction and traffic prediction. Despite their critical importance, time-series datasets frequently encounter missing values due to various factors, including sensor or equipment malfunctions, privacy constraints, and technological limitations. For instance, in single-cell RNA sequencing (scRNA-seq) data, the missing rate can range from 50% to 90% [13] due to the limitation of the scRNA-seq technique, depending on the experimental setup and preprocessing methods. Such high proportions of missing values will introduce bias, severely degrade data quality, and undermine the reliability and accuracy of analyses. For example, reconstructing dynamic regulatory networks from time-series genomics data becomes particularly challenging if the time-series scNRA-seq data contains a large amount of missing values. Therefore, the imputation of missing values is a crucial preprocessing step for downstream analyses in scRNA-seq data and the development of different time-dependent analytical models.

The imputation of missing values in high-dimensional time-series data is a challenging problem in statistics and machine learning research. Various imputation techniques have been proposed to tackle the challenge of missing values in time-series data. Early traditional statistics approaches often relied on simplistic strategies, such as deleting records with missing values or replacing them with summary statistics like the mean, median, mode, or the last observed value, or using a autoregressive model, like the ARIMA model [14], which has been used to impute the high-resolution temporal climate time-series data [2]. Apparently, these methods fail to capture the underlying patterns in the data, often leading to biased or inaccurate results. Some machine learning-based approaches, such as Expectation–Maximization (EM)-based algorithms, K-Nearest Neighbors (KNN) [15], Multivariate Imputation by Chained Equations (MICE) [16], matrix factorization techniques [17], and MissForest [18], work under various assumptions. For instance, KNN imputes missing values by identifying the K-Nearest Neighbors based on similarity and estimating the missing entries as a weighted average of the neighbors’ values, while MICE iteratively models the missing data as a function of other variables. Matrix factorization methods assume a low-rank structure in the data matrix, and MissForest uses random forests to predict missing entries. While these methods can be effective for some linear time-series data, they fail to capture the non-linear temporal dependencies and intricate correlations among variables. As a result, these approaches are less effective in handling the dynamic and interconnected nature of complex time-series datasets.

Recent advancements in deep learning, for example, variational autoencoders (VAE) [19], and generative adversarial networks (GAN) [20], have introduced novel strategies for time-series imputation. The VAE-based imputation methods, such as GP-VAE [21], have been applied to address missing values in various datasets, including traffic prediction [22]. However, these methods typically require a complete dataset for training, which can limit their applicability in scenarios with significant amounts of missing data. GAN is another widely used generative model through adversarial training described by a minimax game between a generator and discriminator, which can generate synthetic data following the same distribution as the real data from a random noise; hence, this method has gained significant attention in the field of missing values imputation. Yoon et al. introduced generative adversarial imputation networks (GAINs) [23], implemented by convolutional neural networks (CNNs), to impute missing values in static datasets. Building on this, our recent work developed sc-fGAIN [24], an extension of GAINs, to impute missing values in static single-cell RNA sequencing (scRNA-seq) data using f-divergence as the adversarial loss.

The correlation between different features plays a crucial role in time-series data. Recurrent neural networks (RNNs), known for their ability to model sequential data, are particularly effective at capturing both inter-feature correlations and temporal dynamics, making them well-suited for time-series data imputation. Che et al.’s work, GRU-D [6], is a RNN-based method to impute missing value. In [25], a two-stage Wasserstein–GAN-based method using unidirectional GRUI was proposed for time-series imputation. Later, an end-to-end generative adversarial network (E2-GAN) was introduced in [26], incorporating an autoencoder within the generator to streamline the imputation process and reduce computational complexity. Ni et al. [27] proposed MBGAN, which combines multi-head self-attention mechanisms with bidirectional RNNs to impute missing values. The inclusion of a temporal attention mechanism enables MBGAN to capture crucial correlations in long sequences. Furthermore, other GAN-based imputation approaches have been developed recently, including the adaptive-learned median-filled deep autoencoder (AM-DAE), the non-autoregressive multi-resolution GAN model (NAOMI) [28], the cue Wasserstein generative adversarial imputation network with gradient penalty (CWGAIN-GP), unsupervised semantic generative adversarial networks (USGANs) [29], ImputeGAN [30], self-attention based time-series imputation methods, for example, SAITS and STING [31,32], diffusion-based methods [33], for example, conditional score-based diffusion models for probabilistic time-series imputation [34], and graph-neural-network-based imputation methods [35].

While these generative adversarial network-based imputation methods [25–27,29,30,36] have introduced different strategies to tackle missing data in time-series datasets, they still have a lot of limitations. Most GAN-based imputation methods still depends on the traditional GAN architecture. That is, most existing imputation methods operate under the assumption of a forward directional generative process, inferring missing values solely from past observations while disregarding future information. So, most approaches overlook the bidirectional dependencies often present in time-series data, potentially limiting the accuracy and robustness of the imputation. Moreover, the traditional GAN often suffers from mode collapse, where the generator produces a limited variety of outputs, failing to capture the full diversity of the target data distribution.

To address the limitations of traditional imputation methods, building on Luo et al.’s work [25] and our recent study [24], we propose a novel approach called the f-divergence-based bidirectional deep generative adversarial imputation network (tf-BiGAIN) for time-series data imputation. This approach uses f-divergence as the adversarial loss to enhance diversity in the learning process, while incorporating a bidirectional gated recurrent unit in the GANs to impute missing values by utilizing both past and future temporal information. Finally, we evaluated the effectiveness and potential limitations of the proposed method by analysing real-world time-series data and comparing it to other imputation methods.

## 2. Materials and Methods

In this section, we will discuss how we extend previous work [24,25] and introduce a novel approach that combines f-divergence and bidirectional gated recurrent unit-based generative adversarial imputation networks to impute missing values in time-series data. We begin by formally defining the problem, reviewing relevant preliminaries on multivariate time-series data, and adopting notations consistent with those in [25].

### 2.1. Problem Setup

Assume a multivariate time-series incomplete dataset, $X=\left\{x_{1},x_{2},\ldots ,x_{i},\ldots ,x_{T}\right\}\in {\mathbb{R}}^{D\times T}$, which consists of a sequence of T observations with some missing entries. Each observation is associated with a timestamp $t_{i}$, where the i-th observation $x_{i}\in {\mathbb{R}}^{D}$ is composed of D features, denoted as $\left\{x_{i}^{1},x_{i}^{2},\ldots ,x_{i}^{d},\ldots ,x_{i}^{D}\right\}$. The set of timestamps for all observations is represented by $T=\left\{t_{1},t_{2},\ldots ,t_{i},\ldots ,t_{T}\right\}\in {\mathbb{R}}^{T}$, with $t_{1}=0$. It is important to note that the intervals between consecutive timestamps $t_{i}$ may vary and are not uniform. For instance, consider the following incomplete matrix, which has T=5 time points and D=3 features. Missing values in the data are denoted by question marks (?), and the timestamps are given by T=(0,3,5,8,12):

$$\left(\begin{array}{ccccc} x_{1} & x_{2} & x_{3} & x_{4} & x_{5}\\ 2 & ? & ? & 5 & 9\\ 3 & 8 & ? & ? & ?\\ ? & 6 & ? & 10 & 1 \end{array}\right)$$

A mask matrix $M\in {\mathbb{R}}^{D\times T}$ is used to indicate the presence or absence of values in $x_{t}$. The entries of M are defined as follows:

$$M_{i,d}=\{\begin{array}{ll} 0 & \mathrm{if}\;x_{i}^{d}\;\mathrm{is missing},\\ 1 & \mathrm{otherwise}. \end{array}$$

This mask matrix provides a binary representation, where 0 denotes missing values, and 1 denotes observed values in the data.

Previous studies [25,37] have emphasized the significance of time lags or gaps between consecutive observed values or valid observations in modeling incomplete time-series data, as these intervals often follow an unknown, non-uniform distribution that directly impacts the underlying data dynamics. A time-lag matrix $\delta \in {\mathbb{R}}^{D\times T}$ is introduced to capture the time lag between consecutive observations for each feature. Our method can process the time-series data in both forward and backward directions, so, a time-lag matrix is constructed separately for each direction. In the forward direction, which processes data from earlier to later time steps, the entries of the forward time-lag matrix, $\delta^{f}$, for the d-th feature are defined as:

(1) $$\delta_{i,d}^{f}=\{\begin{array}{ll} t_{i}-t_{i-1}, & \text{if}\;M_{i-1,d}=1\;\text{and}\;i>1,\\ \delta_{i-1,d}^{f}+t_{i}-t_{i-1}, & \text{if}\;M_{i-1,d}=0\;\text{and}\;i>1,\\ 0, & \text{if}\;i=1. \end{array}$$

The entries of the backward time-lag matrix, $\delta^{b}$, for the d-th feature are defined as follows:

(2) $$\delta_{i,d}^{b}=\{\begin{array}{ll} t_{i+1}-t_{i}, & \text{if}\;M_{i+1,d}=1\;\text{and}\;i<T,\\ \delta_{i+1,d}^{b}+t_{i+1}-t_{i}, & \text{if}\;M_{i+1,d}=0\;\text{and}\;i<T,\\ 0, & \text{if}\;i=T. \end{array}$$

Then, the forward and backward time-lag matrix of the above example can be written as

$$\delta^{f}=\left[\begin{array}{ccccc} 0 & 3 & 5 & 8 & 4\\ 0 & 3 & 2 & 5 & 9\\ 0 & 3 & 2 & 5 & 4 \end{array}\right];\;\delta^{b}=\left[\begin{array}{ccccc} 8 & 5 & 3 & 4 & 0\\ 3 & 9 & 7 & 4 & 0\\ 3 & 5 & 3 & 4 & 0 \end{array}\right].$$

Our goal is to impute the missing values in the incomplete matrix **X** using a generative adversarial network-based method, with the input of **X**, a random noise matrix **Z**, a mask matrix **M**, and a time-lag matrix δ. Next, we will discuss our f-divergence-based bidirectional deep generative adversarial imputation network (tf-BiGAIN) for the imputation of missing values in the time-series data.

### 2.2. Generative Adversarial Imputation Network

Generative adversarial networks (GANs) [20] are designed to model the data distribution (x∼p(x) which is unknown) by training two adversarial neural networks: the Generator (G(θ)) and the Discriminator (D(ϕ)). The Generator aims to transform a noise vector (z∼q(z)) into synthetic samples (G(z,θ)) that closely resemble real samples to deceive the Discriminator. Conversely, the Discriminator’s objective is to distinguish between authentic samples (x) and synthetic ones (G(z,θ)) produced by the Generator. Here, θ and ϕ represent the parameters of the Generator and Discriminator networks, respectively, which are optimized using the Backpropagation algorithm by minimizing cross-entropy loss in the GAN framework [20]. The primary goal of the vanilla GAN is to minimize the Generator’s loss while simultaneously maximizing the Discriminator’s loss. This adversarial process is formulated as a minimax optimization problem:

(3) $$\underset{G}{\text{min}}\;\underset{D}{\text{max}}\;V(D,G)=\underset{\theta }{\text{min}}\;\underset{\phi }{\text{max}}\left[{𝔼}_{x}(\text{log}\;D(x,\phi ))+{𝔼}_{z}(\text{log}(1-D(G(z,\theta ),\phi )))\right].$$

**Figure 1.**
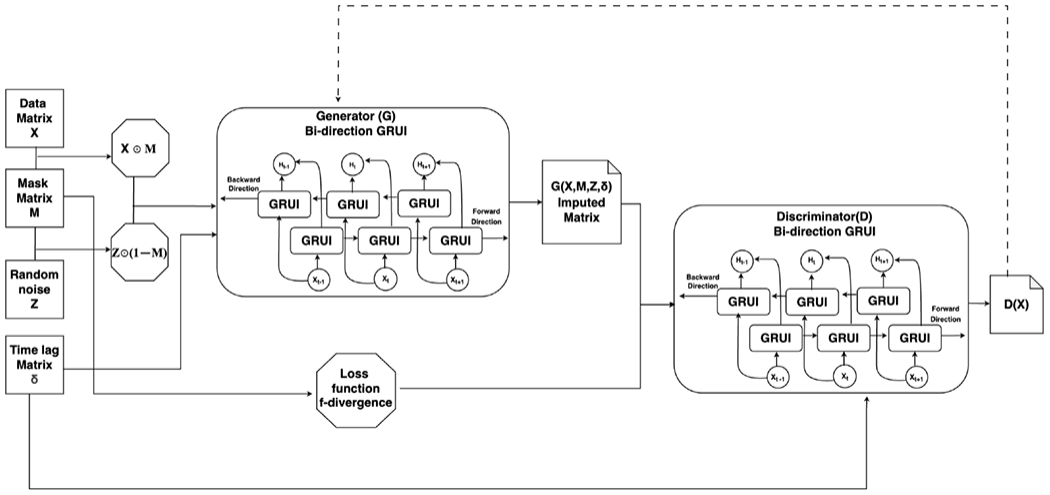
An illustration of the bidirectional f-divergence based generative adversarial imputation network architecture. The Generator takes as input the incomplete time-series data, a corresponding mask matrix, and a time-lag matrix, along with a random matrix, to generate synthetic imputed data. The Discriminator distinguishes between real and imputed values generated by the Generator. Both the Generator and Discriminator are implemented using bidirectional GRUI and trained using f-divergence loss functions.

The proposed f-divergence-based bidirectional deep generative adversarial imputation network (tf-BiGAIN) for time-series data imputation is illustrated in Figure 1. A critical component of the tf-BiGAIN architecture is the Generator (G), which takes as the input the incomplete time-series data matrix X, a random noise matrix Z, a binary mask matrix M, and a time-lag matrix δ. The Generator can produce imputed values for the missing observations. The input to the Generator is formulated as a combination of observed and random values, represented by the element-wise operations between M,X,Z, and (1−M), specifically: M⊙X+(1−M)⊙Z, where the symbol ⊙ represents the Hadamard product (element-wise multiplication) between two matrices of the same dimensions. So, M⊙X retains the observed entries from X, and (1−M)⊙Z fills the missing entries with values generated from the noise matrix Z.

The output of Generator, representing the imputed values, is denoted as G(X,M,Z,δ), and the complete data is defined as $\overset{ˆ}{X}=M⊙X+(1-M)⊙G(X,M,Z,\delta )$, which combines the observed entries from X with the imputed values generated by G. This complete data matrix $\overset{ˆ}{X}$ is then passed as input to the Discriminator network, another critical component of the tf-BiGAIN architecture. The Discriminator’s primary role is to distinguish between real (observed) data and imputed data. It achieves this by predicting the binary mask vector M, indicating which entries are observed and which are generated. This adversarial interaction between the Generator and the Discriminator drives the Generator to produce more realistic imputations, improving the overall imputation performance of the tf-BiGAIN framework.

### 2.3. Bidirectional Gated Recurrent Unit

In static data analysis, generative adversarial network (GAN)-based methods such as GAIN [23] and sc-fGAIN [24] utilized convolutional neural networks (CNNs) to implement the Generator and Discriminator. For time-series data, however, recurrent neural networks (RNNs), for example, long short-term memory (LSTM) [38] network and gated recurrent unit (GRU) [39], are more suitable due to their ability to model sequential data, capture temporal dependencies, and account for correlations between different features. Figure 1 illustrates that, in our tf-BiGAIN architecture, both the Generator and Discriminator are implemented by bidirectional modified GRU (GRUI) and trained using f-divergence loss functions.

**Figure 2.**
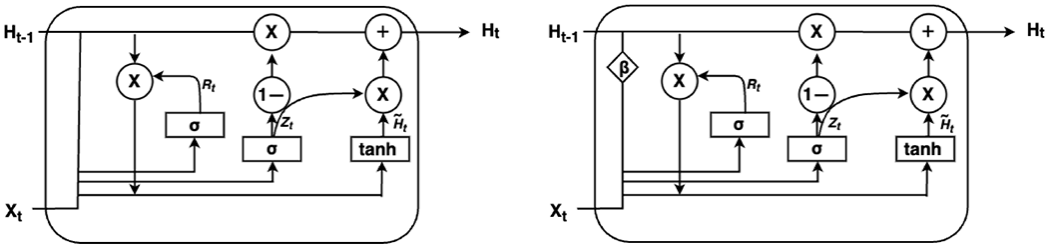
Structure of gated recurrent unit (left) and modified gated recurrent unit (right).

Before introducing the bidirectional GRUI network, we first review the basics of the GRU network. The GRU is a simplified version of the LSTM, designed without a separate memory cell in its architecture. As shown in Figure 2a, the GRU consists of two key gates: the reset gate (***R***) and the update gate (***Z***). The reset gate (***R_t_***), a vector with values in [0, 1], determines how much of the previous hidden state ***H***_*t*−1_ should contribute to the computation of the candidate hidden state (${\tilde{H}}_{t}$). By selectively “resetting” part of the hidden state, the reset gate allows the GRU to focus on recent information from the current input (***X***_*t*_) and discard irrelevant historical context when necessary. The update gate ***Z_t_***, also a vector with values in [0, 1], functions as a “memory controller”; it controls how much of the previous hidden state (***H***_***t***−1_) is carried forward to the current hidden state (***H***_***t***_). So, this mechanism ensures that the GRU can balance stability (long-term memory) and adaptability (response to new inputs), making it well-suited for modeling sequential data.

The reset gate $R_{t}$, candidate hidden state $\left({\tilde{H}}_{t}\right)$, update gate $Z_{t}$, and current hidden state $\left(H_{t}\right)$ are defined as:

$$R_{t}=\sigma (W_{r}X_{t}+U_{r}H_{t-1}+b_{r})$$

$${\tilde{H}}_{t}=\tanh\left(W_{h}X_{t}+R_{t}⊙\left(U_{h}H_{t-1}\right)+b_{h}\right)$$

$$Z_{t}=\sigma (W_{z}X_{t}+U_{z}H_{t-1}+b_{z})$$

$$H_{t}=\left(1-Z_{t}\right)⊙H_{t-1}+Z_{t}⊙{\tilde{H}}_{t},$$

where σ represents a sigmoid activation function ensuring the values of $R_{t}$ and $Z_{t}$ are bounded between 0 and 1, W and U represent the weight matrix, b is a bias term which will be learned, and ⊙ represents the Hadamard product (element-wise multiplication).

In time-series data with missing values, the time lag between consecutive observed values is often irregular and follows a nonuniform distribution. Furthermore, the influence of past observations naturally diminishes over time, especially when a variable has not been observed for a long time. To account for this decaying influence, a time-lag matrix is constructed to record the time intervals between the current value and the most recent observed value, such that the temporal dynamics of the data can be appropriately modeled, even in the presence of irregular time gaps.

To control the influence of previous observations, we adopt the modified gated recurrent unit, known as the GRUI cell, introduced in [25], for updating the hidden state of the GRU. This GRUI cell is designed to handle temporal irregularities in incomplete time-series data by accounting for the diminishing influence of past observations as time gaps increase. Figure 2b illustrates the architecture of the GRUI cell, which incorporates a time decay vector (β), a vector with values in [0, 1], to model the decaying influence of past observations over time. This decay vector is a function of the time-lag matrix δ, and a common choice is an exponential decay function, which is defined as:

$$\beta_{t}=1/e^{\text{max}\left(0,W_{\beta }\delta_{t}+b_{\beta }\right)},$$

where $W_{\beta }$ and $b_{\beta }$ are the weight matrix and bias term, respectively. After the decay vector is obtained, the GRU hidden state $H_{t-1}$ is updated by element-wise multiplying the decay factor β, that is, $H_{t-1}⊙\beta_{t}$. Similar to [25], if we assume the weight W=U, the GRUI equations can be rewritten in a concatenated form, significantly reducing the number of parameters. This assumption allows us to treat the input $X_{t}$ and the hidden state $H_{t-1}$ as a single concatenated input vector, simplifying the computation while retaining the temporal dynamics of the model. The concatenated forms of GRUI equations are expressed as:

(4) $$R_{t}=\sigma (W_{r}[X_{t},H_{t-1}⊙\beta_{t}]+b_{r})$$

(5) $${\tilde{H}}_{t}=\tanh\left(W_{h}\left[X_{t},R_{t}⊙H_{t-1}⊙\beta_{t}\right]+b_{h}\right)$$

(6) $$Z_{t}=\sigma \left(W_{z}\left[X_{t},H_{t-1}⊙\beta_{t}\right]+b_{z}\right)$$

(7) $$H_{t}=\left(1-Z_{t}\right)⊙\left(H_{t-1}⊙\beta_{t}\right)+Z_{t}⊙{\tilde{H}}_{t}.$$

Luo et al.’s work [25] utilized a forward unidirectional modified gated recurrent unit (GRUI) to implement both the Generator and the Discriminator for imputing missing values in time-series data. However, this forward-directional GRU-based approach relies solely on past observations to impute missing values, ignoring the bidirectional dependencies inherent in time-series data. Recent studies [40–42] have demonstrated the advantages of using bidirectional recurrent neural networks (RNNs) for time-series analysis. These studies indicate that future observations might influence past trends, and vice versa, making bidirectional structures effective for capturing temporal dependencies.

Algorithm 1 illustrates our bidirectional learning procedure to calculate the hidden states. In the bidirectional GRUI, we calculate the hidden states $H_{t}$ by processing the sequence in both the forward and backward directions. The final output $H_{t}$ at each timestep is the mean of the forward hidden state $\left(H_{t}^{f}\right)$ and the backward hidden state $\left(H_{t}^{b}\right)$. So, the bidirectional GRUI architecture can capture the dependencies from both past and future observations, which might enhance the accuracy of time-series imputation, as demonstrated in our real-world data analysis presented in the following section.

**Algorithm 1:** Bidirectional GRUI for hidden state calculation

1: **Input:** Incomplete time-series data $X=X_{1},X_{2},\ldots ,X_{T}$; Forward time lag $\delta^{f}$; Backward time lag $\delta^{b}$.
2: **Initialization**
3: Initialize forward hidden state: $H_{0}^{f}=0$
4: Initialize backward hidden state: $H_{T}^{b}=0$
5: **Forward Process**
6: **for** *t* = 1 to *T* **do**
7: Compute forward hidden state: $H_{t}^{f}={\mathrm{GRUI}}^{f}\left(H_{t-1}^{f},X_{t},\delta_{t}^{f}\right)$
8: **end for**
9: **Backward Process**
10: **for** *t* = *T* to 1 **do**
11: Compute backward hidden state: $H_{t}^{b}={\mathrm{GRUI}}^{b}\left(H_{t+1}^{b},X_{t},\delta_{t}^{b}\right)$
12: **end for**
13: **Combine Forward and Backward States**
14: **for** *t* = 1 to *T* **do**
15: Compute bidirectional hidden state: $H_{t}=\frac{H_{t}^{f}+H_{t}^{b}}{2}$
16: **end for**
17: **Output:** Bidirectional hidden states $\left\{H_{1},H_{2},\ldots ,H_{T}\right\}$.

As discussed earlier, both the Generator and the Discriminator in our tf-BiGAIN imputation framework are implemented using bidirectional GRUI networks. These models are trained via the Backpropagation algorithm, with parameters optimized by minimizing an adversarial loss function. This loss function evaluates the effectiveness of the Generator in producing realistic imputations and the Discriminator in distinguishing between real and imputed data, thereby driving the optimization process towards improved imputation quality. Traditional vanilla GAN-based methods [23,40] have commonly employed cross-entropy loss [20]; though effective in some scenarios, but these methods suffer from some challenges. One major issue is mode collapse, where the Generator produces limited variations of samples, failing to capture the full diversity of the true data distribution. This limitation is often attributed to the use of binary cross-entropy loss. In contrast, Luo et al. [25] utilized the Wasserstein distance [43] to address some of these challenges in their model training. Our recent work [24] has demonstrated that f-divergence [44] serves as a robust alternative adversarial loss function, significantly enhancing the accuracy of imputing missing values in static single-cell RNA sequencing data. The f-divergence-based generative adversarial network (f-GAN) framework has been discussed in previous studies [24,44]. For completeness, we will briefly review the principles of *f*-GAN and f-divergence based adversarial loss in the next section. By extending this approach to time-series data in the tf-BiGAIN framework, we aim to achieve comparable improvements in imputation performance, benefiting from the enhanced flexibility and stability provided by the use of f-divergence as the adversarial loss function.

### 2.4. f-Divergence-Based Adversarial Loss

The f-divergence, also referred to as the Ali–Silvey distances [45], measures the dissimilarity between two probability distributions, *P* and *Q*. It is formally defined as:

(8) $$D_{f}(P\|Q)={𝔼}_{x∼q}\left[f\left(\frac{p(x)}{q(x)}\right)\right]=\int_{\text{Ω}}q(x)f\left(\frac{p(x)}{q(x)}\right)dx,$$

where p(x) and q(x) are two continuous probability density functions of P and Q defined over the domain Ω, and f(u) is a generator function that is proper, lower semicontinuous, and convex, with the property f(1)=0. The f-divergence provides a flexible framework to quantify the divergence between distributions, with various choices of the generator function f(u) corresponding to well-known divergence measures, such as the (forward and backward) Kullback–Leibler (KL) divergence, Pearson divergence, and Jensen–Shannon divergence.

The estimation of f-divergence relies on the convex conjugate of the generator function f. Every convex lower-semicontinuous function f has a convex conjugate function $f^{*}$, which is also known as Fenchel–Legendre transform of f [46]. The convex conjugate is defined as solving a supremum problem, which computes the maximum value of a linear function subtracted by the original convex function; that is,

$$f^{\text{*}}(t)=\underset{u\in {\text{dom}}_{f}}{\text{sup}}\;\{ut-f(u)\}.$$

The convex conjugate $f^{*}(t)$ is also convex and lower-semicontinuous if f(x) is a convex function on ℝ, so it provides a dual perspective of the function f, enabling alternative representations and analysis in convex optimization and f-divergence estimation. Using the conjugate function, we can estimate the lower bound of the f-divergence which is expressed as [44]:

$$D_{f}(P\|Q)\geq \underset{T\in 𝒯}{\text{sup}}\left({𝔼}_{x∼P}[T(x)]-{𝔼}_{x∼Q}\left[f^{\text{*}}(T(x))\right]\right),$$

where *T*(*x*) is any Borel function, which is also called a critic function.

The f-divergences are more general, and can be used in different GAN architectures to offer a flexible and efficient way to measure the distance between probability distributions. Similar to the minimax problem in the vanilla GAN, where the objective function is expressed using cross-entropy in Equation (3), the f-divergence-based generative adversarial network (f-GAN) reformulates the problem as follows:

(9) $$\underset{\theta }{\text{min}}\;\underset{\phi }{\text{sup}}\left({𝔼}_{x∼P}\left[g_{f}\left(S_{\phi }(x)\right)\right]-{𝔼}_{x∼Q(z,\theta )}\left[f^{\text{*}}\left(g_{f}\left(S_{\phi }(x)\right)\right)\right]\right),$$

where Q(z,θ) is the Generator, implemented by a neural network parameterized by θ. It takes a random noise vector z∼p(z) as the input and generates samples of interest x∼Q. The critic (or Discriminator) $T(x)=g_{f}\left(S_{\phi }(x)\right)$ maximizes the divergence by distinguishing real samples from generated samples, where $S_{\phi }(x)$ is the output of the neural network used to approximate the critic, and $g_{f}$ is an output activation function specific to the choice of f-divergence.

Using the Equation (9), we can obtain a general f-divergence based adversarial loss function for both Generator and Discriminator for training and modeling diverse applications by selecting the most suitable *f* functions [44], for example, the divergence of forward Kullback–Leibler (KL), reverse KL, Jensen–Shannon (JS), Pearson, etc.

To train the Discriminator *D*, the Generator *G* is fixed, and the Discriminator is optimized by maximizing the following f-divergence based objective function:

$${ℒ}_{Df}={𝔼}_{x∼P}\left[g_{f}\left(S_{\phi }(x)\right)\right]-{𝔼}_{x∼Q(z,\theta )}\left[f^{\text{*}}\left(g_{f}\left(S_{\phi }(x)\right)\right)\right].$$

After updating the Discriminator D, the next step is to optimize the Generator G, whose optimization depends on two key loss functions: the reconstruction loss $\left({ℒ}_{R}\right)$ for the observed entries $\left(M_{i,d}=1\right)$ and adversarial loss $\left({ℒ}_{GAf}\right)$ for the missing entries $\left(M_{i,d}=0\right)$. The reconstruction loss measures how closely the generated values for the observed entries $\left(M_{i,d}=1\right)$ match the original observed values. Similar to previous work [23–25], we use the Mean Squared Error (MSE) to calculate the reconstruction loss:

$${ℒ}_{R}=\|X⊙M-G(X,M,Z,\delta )⊙M{\|}^{2},$$

where X is the original data matrix, G(X,M,Z,δ) represents the generated (imputed) data, and M is a binary mask matrix indicating observed entries ($M_{i,d}=1$).

The adversarial loss ensures the Generator produces realistic imputed values for the missing entries ($M_{i,d}=0$) that the Discriminator cannot easily distinguish from real values, so it can closely mimic the true data distribution. The adversarial loss for the Generator, using f-divergence, is defined as:

$${ℒ}_{GAf}=-{𝔼}_{x∼Q(z,\theta )}\left[f^{\text{*}}\left(g_{f}\left(S_{\phi }(x)\right)\right)\right].$$

To achieve a balance between reconstruction accuracy and data distribution fidelity, the overall objective function for the Generator $\left({ℒ}_{Gf}\right)$ is defined as a weighted sum of the reconstruction loss $\left({ℒ}_{R}\right)$ and the adversarial loss $\left({ℒ}_{GAf}\right)$:

(10) $${ℒ}_{Gf}={ℒ}_{GAf}+\lambda {ℒ}_{R},$$

where λ is a hyperparameter that is tuned to balance the trade-off between reconstruction accuracy and the fidelity of the imputed data to the true distribution. Therefore, the Generator is trained by minimizing the objective function in Equation (10).

Compared with previous work [25,40,47], the proposed imputation method introduces two key novelties. First, the bidirectional GRUI network replaces the unidirectional RNN network, enabling the model to leverage both past and future information, which improves its ability to capture bidirectional dependencies in time-series data. Second, an f-divergence-based objective function is used to train the model, providing greater flexibility in optimizing the imputation process and helping address the mode-collapse problem that is often encountered with other loss functions. This allows the framework to adapt to different divergence measures and improve stability during training.

## 3. Results

In this section, we implement the time-series f-divergence-based bidirectional generative adversarial imputation method (tf-BiGAIN), which builds upon the work of Luo et al. [25], to impute missing values in time-series data. To evaluate the effectiveness of our approach, we compared its performance with other state-of-the-art time-series imputation methods on two real-world benchmark datasets: a healthcare dataset and an air quality dataset. These datasets have been widely used as benchmarks in previous imputation studies [6,25,40,41], providing a reliable basis for performance comparison.

### 3.1. Datasets

#### PhysioNet Data:

PhysioNet is a publicly available electronic medical record dataset from the PhysioNet Challenge 2012 introduced by Silva et al. [48], which contains 4000 multivariate time-series clinical records collected from patients in intensive care units (ICUs). Each set of time-series data contains 41 variables, including features such as age, weight, heart rate, glucose levels, and other physiological measurements which were irregularly sampled at the first 48 h after admission to ICU. One goal using PhysioNet data is to predict in-hospital mortality and test the performance of different classifiers, which is a binary classification problem. Since the PhysioNet dataset is very sparse and has around 80% missing values, we will apply our imputation method to impute the missing values and apply the RNN classifier to predict the mortality; the Area Under the Receiver Operating Characteristic Curve (AUC-ROC) score will be used as the primary evaluation metric, assessing the model’s ability to distinguish between patients who survive and those who do not.

#### Air Quality Data:

The air quality dataset is a publicly available dataset from the *KDD CUP Challenge 2018* [49]. This dataset provides historical air quality data from Beijing, including hourly records from weather observatories between 30 January 2017 and 31 January 2018. Each record contains measurements for 13 variables, including PM2.5 (μg/m^3^), PM10 (μg/m^3^), CO (mg/m^3^), weather conditions, temperature, and other related factors. We define a new variable, air quality, which is labeled as “polluted” or “not polluted”. Air pollution is defined based on thresholds: PM2.5 exceeds 75 μg/m^3^, PM10 exceeds 150 μg/m^3^, or NO_2_ exceeds 80 μg/m^3^. If none of these thresholds are exceeded, the air is classified as “not polluted”. The goal is to predict air quality using the remaining 10 variables as features in the models. The outcome variable is binary (polluted vs. not polluted), and the AUC score is used as the evaluation metric to assess the models’ performance. Although the original dataset has a missing rate of less than 2%, we artificially introduce missing rates ranging from 10% to 50% to evaluate the imputation performance of different methods under varying levels of missing data.

### 3.2. Configuration and Experimental Design

To ensure a fair comparison with the unidirectional GAN-based imputation method in the previous work [25], we adopted the same training configurations in our tf-BiGAIN architecture for the PhysioNet data imputation. Specifically, the batch size was set to 128, the input dimension corresponding to the number of predictor variables was 41, and the number of hidden units in the GRUI for both the Generator (*G*) and Discriminator (*D*) was 64. The dimension of the random noise vector was also 64. The model was pretrained for 5 epochs, followed by 30 training epochs. The hyperparameter λ, which balances the reconstruction loss and adversarial loss, was set to 0.15, and the learning rate was fixed at 0.001. The imputation loss is optimized over 400 iterations. These configurations align with those used in [25], enabling a direct performance comparison between the proposed bidirectional tf-BiGAIN and the unidirectional GAN-based imputation method. The settings for the KDD data analysis are largely the same as those used for the PhysioNet data, with a few differences. Specifically, the input dimension is 10, the pretraining epoch is set to 2, and the learning rate is 0.01.

Our study aims to examine the impact of bidirectional imputation and f-divergence adversarial loss on the performance of the imputation model. To ensure a fair comparison with previous methods, we employ an RNN classifier in our analysis, as prior studies [6,25,41,50] have demonstrated that the RNN classifier consistently delivers superior performance in imputation tasks. In both the PhysioNet and KDD data analyses, we compute the AUC scores using various f -divergences within both unidirectional and bidirectional GRUI-based generative adversarial imputation network architectures. For the KDD dataset, we further evaluate the imputation performance by artificially introducing missing rates ranging from 10% to 50%, allowing us to compare the effectiveness of unidirectional and bidirectional imputation methods under different f-divergence configurations.

### 3.3. Experimental Results

**Figure 3.**
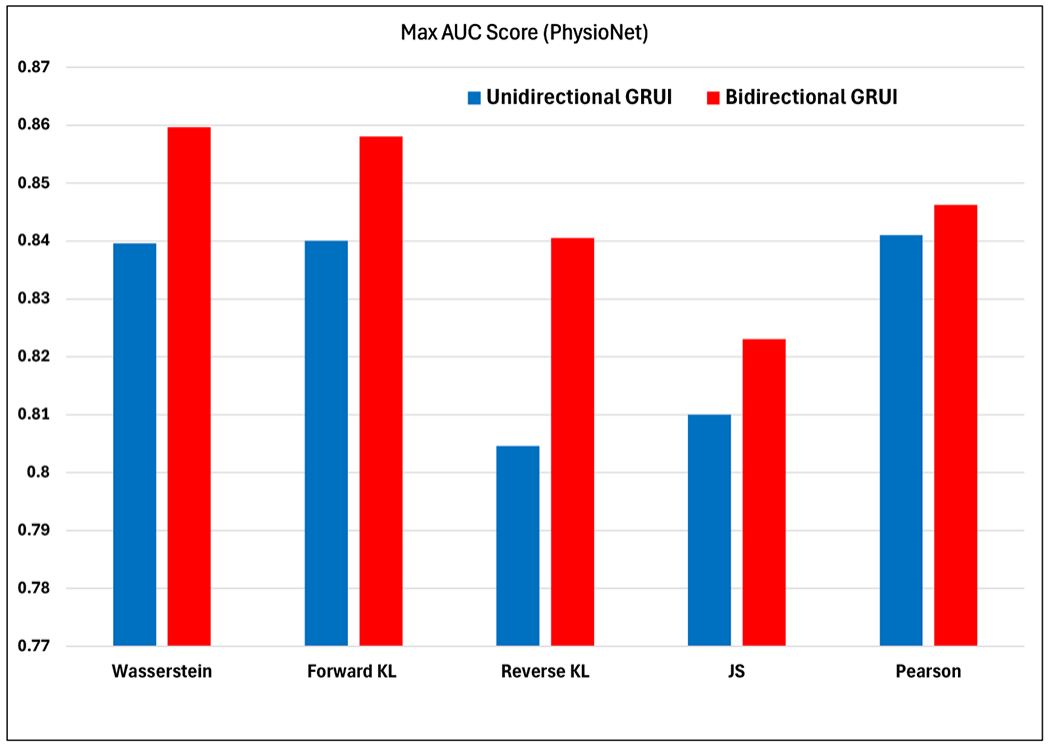
AUC scores for mortality prediction in PhysioNet data using an RNN classifier trained on unidirectional and bidirectional GRUI-based generative adversarial imputation networks with various f-divergence functions as the adversarial loss.

Figure 3 illustrates the AUC scores for mortality prediction using the PhysioNet data from one experimental run. The prediction model is based on an RNN classifier trained on unidirectional and bidirectional GRUI-based generative adversarial imputation networks with various f-divergence functions, including forward Kullback–Leibler (KL), reverse KL, Jensen–Shannon (JS), Pearson, and Wasserstein divergence as the adversarial loss. These results demonstrate the performance of unidirectional and bidirectional GRUI-based GANs with different f-divergence functions in the imputation process. In the unidirectional GRUI-based results, the forward Kullback–Leibler (KL) and Pearson divergence yield the best performance, with AUC scores exceeding 0.84. Luo et al.’s results [25] show that their method performs second best to forward KL and Pearson divergence, which suggests that the choice of adversarial loss function significantly impacts the prediction ability of the model. This finding underscores the importance of selecting an appropriate f-divergence for optimizing the imputation model’s performance.

Table 1 summarizes the AUC score ranges achieved by various baseline imputation methods applied to the PhysioNet data for mortality prediction, as reported in previous studies [25,41], which include mean/zero imputation, logistic regression, MICE, and SVM-based non-RNN imputation methods, as well as four recurrent neural network-based imputation methods: GRU-simple/forward/mean, GRU-D, M-RNN, and BRITS. The non-RNN-based imputation methods demonstrate relatively poor performance, with AUC scores ranging from 0.5 to 0.83. In contrast, the RNN-based imputation methods show significantly better performance, with AUC scores ranging from 0.81 to 0.85. This comparison underscores the advantages of leveraging recurrent neural networks for imputing missing values in time-series data.

Our results in Figure 3 demonstrate a notable improvement in AUC scores, with bidirectional GRUI across all divergence measures compared to the unidirectional GRUI results. Among the various divergence functions, forward KL and Wasserstein divergence-based bidirectional GRUI achieve the best performance, setting a new state of the art for mortality prediction; with AUC scores close to 0.86, our methods outperform all existing baseline imputation methods, as shown in Table 1. This improvement highlights the effectiveness of bidirectional GRUI and the choice of divergence functions in missing value imputation and enhancing predictive accuracy.

**Figure 4.**
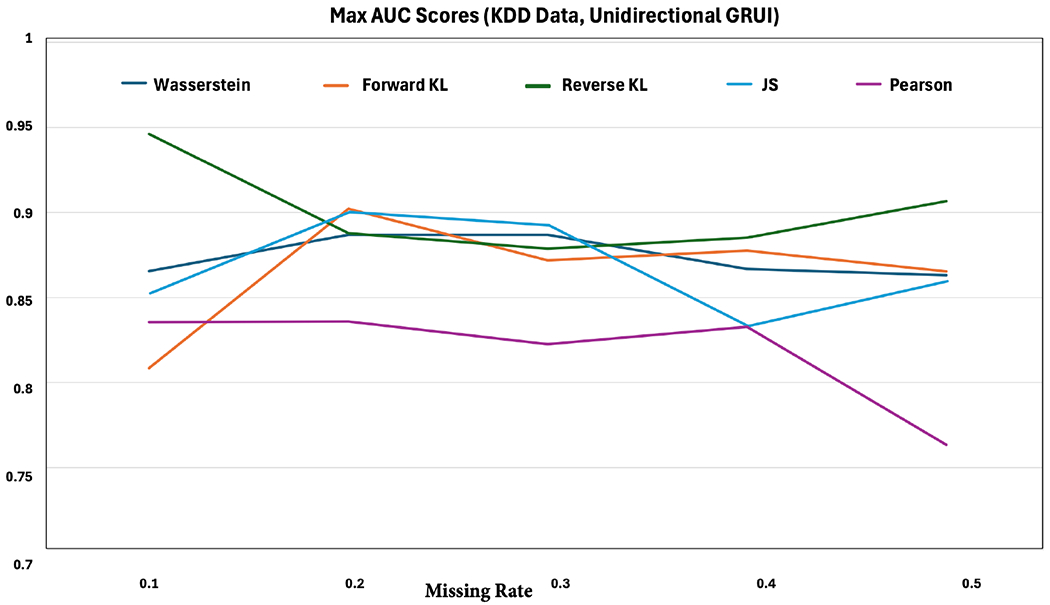
AUC scores for air pollution prediction in KDD air quality data using an RNN classifier trained on unidirectional GRUI-based generative adversarial imputation networks with various f-divergence functions as the adversarial loss across varying missing rates.

Next, we apply both unidirectional and bidirectional GRUI-based f-divergence-based generative adversarial imputation methods to impute the air quality data from the KDD CUP Challenge 2018 and predict whether the air is polluted or not. Figure 4 presents the AUC scores for pollution prediction using unidirectional GRUI-based imputation methods across varying missing rates from one experimental run. To simulate missingness, we randomly remove data points at specified proportions. Our results show that, within the unidirectional GRUI framework, most divergence functions achieve comparable performance in missing value imputation; specially, the forward KL and reverse KL divergence consistently achieve the best performance, outperforming Luo’s method [25], even at high missing rates. These findings demonstrate the robustness and flexibility of f-divergence in imputing missing data and enhancing prediction accuracy under challenging conditions.

**Figure 5.**
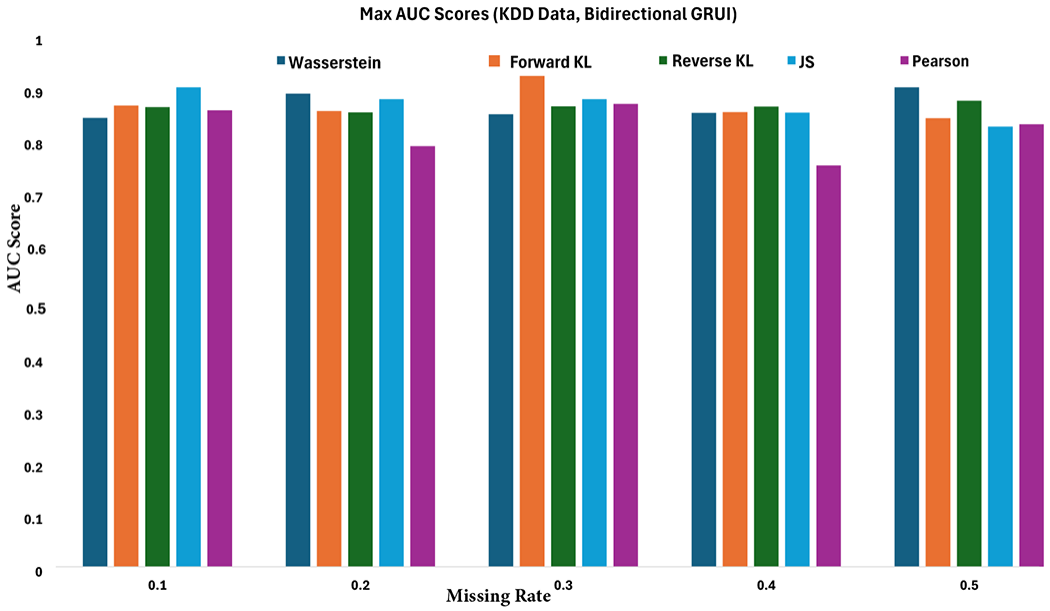
AUC scores for air pollution prediction in KDD air quality data using an RNN classifier trained on bidirectional GRUI-based generative adversarial imputation networks with various f-divergence functions as the adversarial loss across varying missing rates.

Figure 5 illustrates the AUC scores for pollution prediction achieved by the bidirectional GRUI-based generative adversarial imputation method using various f-divergence measures across different missing rates. Table 2 provides the mean AUC scores and their standard errors for different divergence measures and missing rates, based on five experimental runs. The results demonstrate that, within the bidirectional framework, the imputation methods exhibit strong robustness to varying missing rates, with most f-divergence measures delivering excellent prediction performance. These findings highlight the bidirectional approach’s ability to effectively utilize information from both past and future observations, thereby enhancing the model’s capacity to impute missing values and predict outcomes accurately.

## 4. Discussion

In this work, we have developed a novel f-divergence-based bidirectional and undirectional generative adversarial imputation network method, called tf-BiGAIN, to impute missing values in time-series data. Unlike traditional imputation methods, tf-BiGAIN employs a generative model to synthesize missing values without relying on distributional assumptions. The imputation is achieved by training two neural networks, implemented using either bidirectional or undirectional GRUI architectures, with f-divergence serving as the objective function to guide the model’s optimization.

Compared to existing deep learning-based methods, our approach introduces two key novelties. First, f-divergence-based adversarial loss: it extends our previous static missing value imputation method, sc-fGAIN [24], to time-series data, providing a flexible and robust framework for imputation. Second, bidirectional GRUI architecture: by adopting and extending Luo et al.’s GRUI framework [25], our model utilizes both forward and backward temporal information, effectively capturing dependencies from both past and future observations. These innovations significantly enhance the accuracy of time-series imputation. We applied tf-BiGAIN to two real-world time-series datasets, demonstrating its superior performance in predicting mortality and air pollution compared to baseline imputation methods. Moreover, the incorporation of f-divergence provides a flexible and adaptable way to optimize the model for diverse imputation tasks, so, the proposed tf-BiGAIN method is more versatile compared to other imputation methods.

Despite its promising potential, tf-BiGAIN has some limitations that should be acknowledged. Currently, missing values are represented by zeros; however, some zeros in the dataset are real observations rather than missing values. Our method cannot distinguish between real observed zeros and missing values, which may affect imputation accuracy. To address this limitation, we plan to develop new methods in future studies to improve the algorithm’s ability to impute technical zeros while preserving true observed zeros. Moreover, the proposed imputation method requires more temporal observations for effective model training, which is a fundamental requirement for generative approaches. In future work, we aim to explore ways to extend our method to better handle datasets with a limited number of time points. Recently, diffusion models, another class of generative methods, have been applied to impute missing values [33,34,51,52], demonstrating promising results. We plan to explore how to integrate diffusion models with generative adversarial networks to enhance the imputation of time-series data. In the long term, we aim to integrate our imputation methods with the change-point detection algorithm [53] to reconstruct time-varying networks from time-series single-cell RNA sequencing data, which often contains significant amounts of missing values. In conclusion, our findings highlight the promising potential of tf-BiGAIN for time-series data imputation across various domains, paving the way for more accurate and robust analyses in fields such as healthcare, environmental science, and genomics.

## Figures and Tables

**Table 1.** Summary of AUC scores for various imputation methods applied to the PhysioNet dataset, as reported in previous studies.

Method	AUC Score Ranges [25,41]
Mean/Zero	0.5 ~ 0.75
Logistic Regression	0.74 ~ 0.76
SVM	0.81 ~ 0.83
GRU-simple/forward/mean	0.81 ~ 0.82
GRU-D	0.834 ~ 0.842
MICE	0.7642
M-RNN	0.817
BRITS	0.85

**Table 2.** Comparison of AUC scores (mean ± standard error) for bidirectional GRUI-based generative adversarial imputation method with different f-divergences across various missing rates based on five experimental runs.

Missing Rate	Wasserstein	Forward KL	Reverse KL	JS	Pearson
0.1	0.857 ± 0.07	0.859 ± 0.04	0.881 ± 0.02	0.876 ± 0.01	0.858 ± 0.03
0.2	0.875 ± 0.01	0.864 ± 0.04	0.841 ± 0.02	0.878 ± 0.03	0.879 ± 0.03
0.3	0.855 ± 0.02	0.852 ± 0.03	0.856 ± 0.03	0.874 ± 0.01	0.878 ± 0.04
0.4	0.872 ± 0.03	0.877 ± 0.03	0.840 ± 0.03	0.858 ± 0.04	0.857 ± 0.03
0.5	0.853 ± 0.05	0.867 ± 0.03	0.848 ± 0.03	0.895 ± 0.02	0.863 ± 0.04

## Data Availability

The original PhysioNet Challenge 2012 data presented in the study are openly available in PhysioNet at https://physionet.org/content/challenge-2012/1.0.0/ (accessed on 16 December 2024). The original KDD CUP 2018 data are openly available in Monash Time Series Forecasting Repository at https://doi.org/10.5281/zenodo.4656719 (accessed on 16 December 2024). The Python code for the proposed method is available on GitHub: https://github.com/wenshanliu-slu0/Bidirectional_f-Divergence-Based_GAN_Imputation (accessed on 16 December 2024).

## Abbreviations

The following abbreviations are used in this manuscript:

GAN: Generative adversarial network
RNN: Recurrent neural network
GRU: Gated recurrent unit
AUC: Area under the curve
